# Evaluation of psychometric properties of the Persian version of the predicaments questionnaire, exploring social attitudes to suicide

**DOI:** 10.3389/fpubh.2022.1061673

**Published:** 2023-01-06

**Authors:** Ali Rafati, Leila Janani, Seyed Kazem Malakouti, Seyed Abbas Motevalian, Ali Kabiri, Yeganeh Pasebani, Mohammadreza Shalbafan

**Affiliations:** ^1^School of Medicine, Iran University of Medical Sciences, Tehran, Iran; ^2^Preventive Medicine and Public Health Research Centre, Psychosocial Health Research Institute (PHRI), Department of Biostatistics, School of Public Health, Iran University of Medical Sciences, Tehran, Iran; ^3^Mental Health Research Center, Tehran Institute of Psychiatry–School of Behavioural Sciences and Mental Health, Iran University of Medical Sciences, Tehran, Iran; ^4^Research Center for Addiction and Risky Behaviors (ReCARB), Psychosocial Health Research Institute (PHRI), Department of Epidemiology, Iran University of Medical Sciences, Tehran, Iran; ^5^Mental Health Research Center, Psychosocial Health Research Institute (PHRI), Department of Psychiatry, School of Medicine, Iran University of Medical Sciences, Tehran, Iran

**Keywords:** psychometrics, reproducibility of results, surveys and questionnaires, suicide, mental health

## Abstract

**Background:**

Due to the growing prevalence of suicide, assessing people's attitudes toward suicide is necessary. Therefore, this study aimed to examine the psychometric properties of the Persian version of the Predicaments Questionnaire (PQ), measuring social attitudes toward suicide.

**Methods:**

This psychometrics study evaluated face validity, content validity, temporal stability, internal consistency, and construct validity. First, the questionnaire was translated into Persian by the translate-back-translate method. The Persian version was provided to 10 experts in psychiatry for further revision. Two indicators, CVR and CVI, were calculated to evaluate the content validity. To check the face validity, we prepared a form and gave it to 10 people outside the campus to submit their opinions. Temporal stability was investigated by the test-retest method, reporting Intraclass correlation (ICC). Internal consistency was assessed by reporting Cronbach's alpha and McDonald's Omega coefficients. Construct validity was assessed using the confirmatory factor analysis to determine the number of dimensions of the questionnaire.

**Results:**

A total of 151 students were enrolled with a mean age of 25 (SD = 0.32). The Persian PQ was valid in terms of content validity and face validity. Furthermore, it was reliable as Cronbach's alpha, McDonald's Omega, and the ICC were 0.94, 0.943, and 0.998, respectively. In addition, the confirmatory factor analysis yielded one dimension. Finally, after reviewing the experts' comments, the final amendments were made, and only question 29 was removed from the final version.

**Conclusion:**

Consequently, the Persian version of the PQ is acceptable in terms of content validity, face validity, temporal stability, and internal consistency.

## Background

Suicide means deliberately taking one's own life, and if it is complete, it means a deadly act to achieve one's desire for death (1). Every 40 seconds, one person in the world dies due to suicide, which numbers 800,000 a year. Suicide is known as the second leading cause of death between the ages of 15 and 29. For every suicide resulting in death, more than 20 others attempt suicide (2). The suicide rate in 2012 in the world was estimated to be 12 out of every 100,000, accounting for 793,000 deaths in 2016 (3). This amount was 5.3 per 100,000 for Iran (4, 5). Approximately 1.4% of all deaths result from suicide worldwide (3, 6). The rate of complete suicide is higher in men than women, ranging from 1.5 times higher in developing countries to 3.5 times higher in developed countries. Suicide is generally prevalent among people over 70 years of age. However, in some countries, people between 15 and 30 are at higher risk (2). Ten to 20 million non-lethal suicides occur each year (7). Non-lethal suicide attempts can lead to long-term injuries and disabilities. In the Western world, there are more suicide attempts among young people and women (8). Common methods of suicide include hanging, pesticide poisoning, and weapons (9).

The suicide rate in Iran is lower than the world average, still higher than the Middle East average. The most prevalent method of suicide in Iran is hanging in men and burning in women (4, 10). Although women attempt more suicides than men, they attempt fewer completed suicides than men, reflecting the difference in the method of suicide between them (11).

In Iran, the highest suicide rate for men is observed in Lorestan, Hamedan, and Ilam provinces and for women in Lorestan and Kermanshah provinces (10). As a result, Ilam, Kermanshah, Lorestan, and Hamedan provinces have the highest suicide mortality rates in Iran. One reason for such a high suicide rate might be due to the low socioeconomic status. On the other hand, these provinces have the country's highest unemployment and divorce rates. Finally, the tribal structure and extreme fanaticism could be other potential reasons (12).

The most common risk factors for suicide are mental illnesses (including depression, bipolar disorder, autism, schizophrenia, personality disorders, anxiety disorders, and substance abuse), relationship problems, employment and financial hardships, and history of suicide attempts (13–15). However, certain suicides are impulsive and abrupt reactions to stress, marital problems, or rape. People with a history of suicide are at higher risk of attempting suicide. Practical suicide prevention efforts include accurate media coverage of suicide, economic improvement, and restricting access to weapons, poisons, and drugs. Common methods of suicide vary in different regions and countries and depend on the availability of these methods in these regions (16).

Several studies have assessed the attitudes toward suicide in an attempt to explore the underlying factors affecting people's attitudes. For instance, country and cultural differences and socioeconomic status were sources of attitude differences toward suicide (17–19). Also, sex plays a crucial role, as women were observed to have more understanding attitudes toward suicide than men (17, 19). It has been shown that broad existential themes, such as religion, honor, and life's meaning, have influenced perspectives on suicide (20). The Abrahamic religions, for instance, traditionally consider suicide a crime against God, believing in life's sanctity. In this regard, in some countries, suicide is widely regarded as a criminal offense (21). In the 20^th^ and 21^st^ centuries, suicide has rarely been utilized as a means of protest. Moreover, suicide bombings as a terrorist tactic have been observed (22). Suicide is often considered a major disaster and is regarded as an adverse action almost globally.

Community attitudes toward suicide play a vital role in one's desire to attempt suicide. Negative attitudes, by imposing weakness, shame, sin, or guilt on individuals, stigmatize suicide, leading them to consider suicide as a definitive option. In contrast, positive attitudes, by reducing stigma, increase people's help-seeking and, therefore, diminish suicide occurrence (23). It has been proposed that people's attitude to suicide depends heavily on their emotional closeness to the person considering attempting suicide (24). For instance, nurses and practitioners who handle suicidal patients, compared to those who do not, show a more positive (accepting) attitude to suicide (25).

Numerous studies have examined communities' attitudes toward suicide (26). A major challenge in measuring people's attitudes toward suicide is their subjectivity and variability over time. There are certain instruments and scales that intend to assess people's attitudes toward suicide but some are designed for a particular social group of people, for instance military (23) or nursing personnel (25). A significant number of instruments contain suicide myths, i.e., society's misconceptions about suicide. According to the World Health Organization, one of these myths and misconceptions is the belief that “only people with mental disorders attempt suicide.” Other myths include: Talking about suicide encourages it; most suicides occur without warning; people who attempt suicide do not talk about it; people who attempt suicide are determined to die (27).

The Predicaments Questionnaire designed by Shahtahmasebi et al. in December 2016 measures the social attitude toward suicide in all individuals, regardless of any social groups or socioeconomic classes. It also removes the questionnaire from suicide myths. This study considers suicide as a response to predicaments, and it demonstrates that the questionnaire's scores have a direct relationship with the suicide rate in a group or population (28). Multi-center and international studies in this field are highly required. Therefore, this study aimed to examine the psychometric properties of the Persian translation of the “Social Attitudes to Suicide dealing with Predicaments” Questionnaire.

## Methods

This psychometric research was performed at the Iran University of Medical Sciences in 2020. The analysis included face validity, content validity, temporal stability reliability, internal consistency reliability, and construct validity. First, with the questionnaire designer's permission, the questionnaire was received *via* email, and then it was translated into Persian by the translate-back-translate method (29). For this purpose, we recruited two independent translators to translate it into Persian and two other independent translators to translate it again into English. Afterwards, we prepared the questionnaire's initial Persian version by examining the Persian and English translations' semantic compatibility and the original English version. We provided ten experts in psychiatry with the Persian version to complete and modify the questionnaire by reviewing, discussing, exchanging opinions, and checking cultural adaptation.

We calculated two indicators, the Content Validity Ratio (CVR) (30) and the Content Validity Index (CVI) (31), to evaluate the content validity. For obtaining CVR, we asked experts to categorize each question based on the essentialness. For calculating CVI, they specified relevance, clarity, and simplicity for each question. Based on the number of specialists (10 in this study), the minimum accepted CVR score is 0.62. The minimum acceptable score for a CVI is 0.79 (31).

To check the face validity, we prepared a form and gave it to 10 people outside the campus to submit their opinions. The results were then given to 10 experts to comment on. These ten included psychiatrists, psychologists, and epidemiologists from the Iran University of Medical Sciences. We subsequently asked them to score (using the 5-state Likert-type scale of 1 to 5) the importance of each question. Then we calculated the impact score of each question using the following formula: Impact score = Frequency (%) of the experts scoring 4 or 5 × mean score of the question. The minimum acceptable impact score is more than 1.5 (32).

In the reliability analysis, we examined two areas: temporal stability and internal consistency. First, we evaluated the temporal stability by the test-retest method. For this purpose, 30 participants were randomly selected and completed the questionnaire in a pilot study. Then 2 weeks later, they completed it again. We reported the correlation between the two tests' scores using the Intraclass Correlation Coefficient (ICC). The internal consistency is checked by reporting Cronbach's alpha coefficient and McDonald's Omega coefficient. One hundred fifty-one students of the Iran University of Medical Sciences completed the questionnaire. We calculated Cronbach's alpha coefficient and McDonald's Omega coefficient by analyzing the collected data.

We evaluated construct validity by confirmatory factor analysis to confirm the one-dimensionality of the questionnaire. In this analysis, we measured χ*2*, the Root Mean Square Error of Approximation (RMSR), Comparative Fit Index (CFI), Tucker–Lewis index (TLI), and Standardized Root Mean Square Residual (SRMR) (33).

### Research population and sampling method

We performed this study on the Iran University of Medical Sciences students with ten faculty members in psychiatry, psychology, and epidemiology as experts. The experts evaluated the face and the content validities. To check the internal consistency, we selected a random sample of 200 people from the Iran University of Medical Sciences school of medicine. The questionnaire was emailed to or sent to them as a text message, and subsequently, 151 of them responded and filled out the questionnaire. In total 30 of the 151 students refilled the forms 2 weeks later to test-retest.

### Methods and tools of data collection

The study questionnaire, called the Predicament Questionnaire, was prepared by Shahtahmasebi et al. (28). The questionnaire consists of 41 items. The first nine items record demographic information and personal information, including religion, history of suicide, attitudes toward suicide, depression, hopelessness, and lack of interest. The following 32 items describe scenarios and ask whether the person has the right to attempt suicide in those circumstances. The most common predicaments discussed in this study include the onset of a terminal illness, becoming wheelchair-dependent, severe mental disorders, and car crashes while drunk. For each question, the respondent chooses from a Likert-type scale of 1 to 4, of which 1 is the lowest value and 4 is the highest.

Here is a sample of the questions of the studied instrument:

“Person C was driving below the speed limit on a suburban street. A child ran onto the road. To avoid the child, person C swerved and killed an adult on the other footpath. Would person C have suicidal thoughts?

1. No 2. Slight 3. Moderate 4. Strong.”

This questionnaire was shown to measure only one factor. Moreover, it was indicated to have a high Cronbach's Alpha coefficient showing a high internal consistency (28).

### Method of analysis

We Calculated CVR, CVI, impact score, Cronbach's alpha, and ICC in SPSS software version 22 and performed confirmatory factor analysis and McDonald's Omega in R software version 4, using the R packages “effectsize” and “psych”, respectively.

### Ethics approval and consent to participate

We adhered to the Helsinki Statement and the Ethics Committee of the Iran University of Medical Sciences protocols in all stages of the research. Written informed consent was obtained from all participants. All respondents' information was kept confidential by the researchers. This study was approved by the Ethics Committee of the Iran University of Medical Sciences with the ID code IR.IUMS.FMD.REC.1399.033.

## Results

Of the 151 participants, 42 (27.8%) were male, and 109 (72.2%) were female. In terms of age, all participants were in the range of 20 to 30 years old. In terms of marital status, 81 were single (53.6%), 56 were married (37.1%), five were divorced (3.3%), and nine were uncategorised (6%) (Table 1). The frequency of the responses is shown in Table 2. The respondents' mean questionnaire score was 61.98 (SD = 1.45) out of a maximum score of 128. Furthermore, we calculated the mean scores by sex and marital status as follows: 60.78 (SD = 18.02) and 62.45 (SD = 17.89) for men and women, respectively, and 65.53 (SD = 16.12), 58.15 (SD = 19.38), and 54.40 (SD = 14.63) for the single, the married, and the divorced, respectively.

**Table 1:** Demographic data.

**Age, M (SD)**	**25 (SD = 0.32)**
Sex, *n* (%)	Female: 109 (72.2%)
	Male: 42 (27.8%)
Marital status, *n* (%)	Single: 81 (53.6%)
	Married: 56 (37.1%)
	Divorced: 5 (3.3%)
	Others: 9 (6%)

**Table 2:** Frequency of the responses.

**No**.	**Frequency**				**Median**	**Mean (SD)**
	**No (1)**	**Slight (2)**	**Moderate (3)**	**Strong (4)**		
1	82 (54.3%)	40 (26.5%)	26 (17.2%)	3 (2%)	1.00	1.67 (0.83)
2	70 (46.4%)	54 (35.8%)	19 (12.6%)	8 (5.3%)	2.00	1.77 (0.87)
3	77 (51%)	37 (24.5%)	29 (19.2%)	8 (5.3%)	1.00	1.79 (0.93)
4	78 (51.7%)	39 (25.8%)	28 (18.5%)	6 (4%)	1.00	1.75 (0.90)
5	48 (31.8%)	36 (23.8%)	44 (29.1%)	23 (15.2%)	2.00	2.28 (1.10)
6	25 (16.6%)	31 (20.5%)	39 (25.8%)	56 (37.1%)	3.00	2.83 (1.14)
7	27 (17.9%)	25 (16.6%)	35 (23.2%)	64 (42.4%)	3.00	2.90 (1.21)
8	28 (18.5%)	21 (13.9%)	44 (29.1%)	58 (38.4%)	3.00	2.87 (1.21)
9	49 (32.5%)	25 (16.6%)	34 (22.5%)	43 (28.5%)	3.00	2.50 (1.21)
10	73 (48.3%)	43 (28.5%)	30 (19.9%)	5 (3.3%)	2.00	1.78 (0.88)
11	74 (49.0%)	44 (29.1%)	25 (16.6%)	8 (5.3%)	2.00	1.78 (0.91)
12	86 (57.0%)	44 (29.1%)	21 (13.9%)	0 (0%)	1.00	1.57 (0.73)
13	88 (58.3%)	36 (23.8%)	23 (15.2%)	4 (2.6%)	1.00	1.62 (0.84)
14	105 (69.5%)	29 (19.2%)	15 (9.9%)	2 (1.3%)	1.00	1.43 (0.73)
15	98 (64.9%)	26 (17.2%)	20 (13.2%)	7 (4.6%)	1.00	1.58 (0.89)
16	25 (16.6%)	38 (25.2%)	51 (33.8%)	37 (24.5%)	3.00	2.66 (1.03)
17	93 (61.6%)	36 (23.8%)	15 (9.9%)	7 (4.6%)	1.00	1.58 (0.85)
18	28 (18.5%)	30 (19.9%)	51 (33.8%)	42 (27.8%)	3.00	2.71 (1.07)
19	126 (83.4%)	16 (10.6%)	8 (5.3%)	1 (0.7%)	1.00	1.23 (0.57)
20	67 (44.4%)	53 (35.1%)	21 (13.9%)	10 (6.6%)	2.00	1.83 (0.91)
21	129 (85.4%)	16 (10.6%)	6 (4.0%)	0 (0%)	1.00	1.19 (0.48)
22	100 (66.2%)	33 (21.9%)	11 (7.3%)	7 (4.6%)	1.00	1.50 (0.82)
23	60 (39.7%)	43 (28.5%)	34 (22.5%)	14 (9.3%)	2.00	2.01 (1.00)
24	61 (40.4%)	53 (35.1%)	26 (17.2%)	11 (7.3%)	2.00	1.91 (0.93)
25	44 (29.1%)	39 (25.8%)	40 (26.5%)	28 (18.5%)	2.00	2.34 (1.09)
26	37 (24.5%)	32 (21.2%)	36 (23.8%)	46 (30.5%)	3.00	2.60 (1.16)
27	64 (42.4%)	38 (25.2%)	30 (19.9%)	19 (12.6%)	2.00	2.03 (1.06)
28	58 (38.4%)	29 (19.2%)	39 (25.8%)	25 (16.6%)	2.00	2.21 (1.13)
29	116 (76.7%)	14 (9.3%)	12 (7.9%)	9 (6%)	1.00	1.43 (0.876)
30	45 (29.8%)	25 (16.6%)	33 (21.9%)	48 (31.8)	3.00	2.56 (1.22)
31	72 (47.7%)	34 (22.5%)	29 (19.2%)	16 (10.6%)	2.00	1.93 (1.04)
32	90 (59.6%)	34 (22.5%)	23 (15.2%)	4 (2.6%)	1.00	1.61 (0.84)

### Validity results

According to the content validity results, questions 17, 19, 21, 23, 29, 31, and 32 lacked the required CVR score. Furthermore, questions 19, 29, and 32 did not have the required CVI score. Also, all questions were accepted in terms of simplicity and clarity. The average CVI of the questionnaire is 0.95. We assessed face validity using quantitative and qualitative methods. In a quantitative study, we measured each question's importance using a 5-state Likert-type scale, and the impact score of each question was determined. According to the analysis results, questions 4, 19, 21, and 29 lacked the required impact score.

In the qualitative method, we asked experts' opinions about each question. They mentioned the problems, ambiguities, inadequacies in the meaning, and the shortcomings of questions 19 and 20, which had writing problems, and were subsequently modified. Finally, after reviewing the results and discussing the experts' opinions, only question 29 was invalid and removed from the questionnaire. Question 29 not only received low CVI, CVR, and impact score scores but was also deemed unsuitable by the expert panel, as it was decided to be inappropriate in Iranian society. The results of this study can be seen in Table 3.

**Table 3:** Validity results in the Persian version of the predicaments questionnaire.

**No**.		**CVR**	**CVI (Relevance)**	**CVI (Simplicity)**	**CVI (Clarity)**	**Impact score**
1	Person A had a romantic relationship of about 1 year, with person B, but they had not been living together. Person B ended the relationship and commenced a new relationship with a third person. Would person A have suicidal thoughts? 1. No 2. Slight 3. Moderate 4. Strong	1	0.9	1	1	2.73
2	Person A had a romantic relationship with person B of about 1 year, and they had been living together. Person B ended the relationship and commenced a new relationship with a third person. Would person A have suicidal thoughts? 1. No 2. Slight 3. Moderate 4. Strong	1	0.9	1	0.9	3.51
3	Person A and Person B had been married for about 1 year. Person B ended the marriage and commenced a new relationship with a third person. Would person A have suicidal thoughts? 1. No 2. Slight 3. Moderate 4. Strong	0.8	0.8	1	1	1.85
4	Person C was driving below the speed limit on a suburban street. A child ran onto the road. To avoid the child, person C swerved and killed an adult on the other footpath. Would person C have suicidal thoughts? 1. No 2. Slight 3. Moderate 4. Strong	0.7	0.7	0.9	1	1.28
5	Person C attended a party and got drunk. In spite of advice not to drive, and the offer of being driven home by a friend, person C insisted on driving. Person C drove above the speed limit and hit and killed a pedestrian on a pedestrian crossing. Would person C have suicidal thoughts? 1. No 2. Slight 3. Moderate 4. Strong	1	0.9	1	1	3.3
6	Person D has heterosexual intercourse with Person Z. Without Person D's permission, this event is secretly filmed and placed on the web by a third person. Would person D have suicidal thoughts? 1. No 2. Slight 3. Moderate 4. Strong	1	1	1	1	3.87
7	Person D has homosexual intercourse with Person Y. Without Person D's permission, this event is secretly filmed and placed on the web by a third person. Would person D have suicidal thoughts? 1. No 2. Slight 3. Moderate 4. Strong	1	1	1	1	3.96
8	Person E suffers spinal injuries and will be confined to a wheelchair for life. Would person E have suicidal thoughts? 1. No 2. Slight 3. Moderate 4. Strong	1	1	1	1	4.6
9	Person E develops a painful, terminal (will be fatal) disorder. Would person E have suicidal thoughts? 1. No 2. Slight 3. Moderate 4. Strong	1	0.9	1	1	4.6
10	Person F comes from a very high status and well-educated family. Person F is convicted of stealing and jailed. Would person F have suicidal thoughts? 1. No 2. Slight 3. Moderate 4. Strong	0.8	0.8	0.9	0.9	2.22
11	Person F comes from a very high status and well-educated family. Person F studies very hard, but lacks academic skills and at the end of a year at university, fails every subject. Would person F have suicidal thoughts? 1. No 2. Slight 3. Moderate 4. Strong	0.8	1	1	1	3.36
12	Person G comes from an average family. Person G is convicted of stealing and jailed. Would person G have suicidal thoughts? 1. No 2. Slight 3. Moderate 4. Strong	1	0.9	0.7	0.9	2.66
13	Person G comes from an average family. Person G studies very hard, but lacks academic skills and at the end of a year at university, fails every subject. Would person G have suicidal thoughts? 1. No 2. Slight 3. Moderate 4. Strong	0.8	1	1	0.9	3.2
14	Person H and Person X lived in the same street as children and have been life-long, close friends. Person H is killed in a train crash. Would person X have suicidal thoughts? 1. No 2. Slight 3. Moderate 4. Strong	0.8	0.7	1	1	2.04
15	Person H and Person X lived in the same street as children and have been life-long, close friends. Person H kills him/herself by standing in the path of a train. Would person X have suicidal thoughts? 1. No 2. Slight 3. Moderate 4. Strong	0.8	1	1	1	3.28
16	Person J dropped a gas bottle which exploded. Person J sustained severe burns to the face and hands, which left disfiguring scars. Would person J have suicidal thoughts? 1. No 2. Slight 3. Moderate 4. Strong	1	1	1	1	4.5
17	Person K developed a mental disorder which responds well to treatment, and does not cause Person K to lose more than 5 working days per year. Would person K have suicidal thoughts? 1. No 2. Slight 3. Moderate 4. Strong	0.6	0.9	0.9	0.9	2.1
18	Person K develops a mental disorder, which does not respond well to treatment, and Person K is no longer able to work. Would person K have suicidal thoughts? 1. No 2. Slight 3. Moderate 4. Strong	1	1	1	0.9	3.87
19	Person K develops arthritis, which responds well to treatment, and does not cause Person K to lose more than 5 working days per year. Would person K have suicidal thoughts? 1. No 2. Slight 3. Moderate 4. Strong	0.4	0.5	0.8	1	1.12
20	Person K develops arthritis, which does not respond well to treatment, and Person K is no longer able to work. Would person K have suicidal thoughts? 1. No 2. Slight 3. Moderate 4. Strong	0.8	0.8	0.9	0.9	1.75
21	Person L is a great fan of Person M, who is a popular singer, actor and talk-show celebrity. Person M dies when a building collapses. Would person L have suicidal thoughts? 1. No 2. Slight 3. Moderate 4. Strong	0.4	0.7	1	1	1.2
22	Person L is a great fan of Person M, who is a popular singer, actor and talk-show celebrity. Person M dies by jumping from a building. Would person L have suicidal thoughts? 1. No 2. Slight 3. Moderate 4. Strong	1	0.9	0.8	0.9	2.34
23	Person N's parent has committed a serious crime. Person N is aware of the facts. Person N has been subpoenaed to appear in court and will be asked questions under oath, which will probably lead to the parent being convicted and receiving a jail sentence. Would person N have suicidal thoughts? 1. No 2. Slight 3. Moderate 4. Strong	0.6	0.9	0.9	0.9	2.73
24	Person O is in love with Person P, but person O's parents want Person O to marry a third person, of their choosing. Would person O have suicidal thoughts? 1. No 2. Slight 3. Moderate 4. Strong	1	0.9	0.9	0.9	2.8
25	Person Q has a serious gambling problem, has lost the family's savings and is in debt. Bills are starting to arrive which cannot be easily paid. Would person Q have suicidal thoughts? 1. No 2. Slight 3. Moderate 4. Strong	1	1	1	1	3.44
26	Person Q has a serious gambling problem, has lost the family's savings and is deeply in debt. Person Q's family is about to be turned out onto the street by debt collectors. Would person Q have suicidal thoughts? 1. No 2. Slight 3. Moderate 4. Strong	1	0.9	1	1	3.44
27	Person R cannot find work and is having trouble paying the family bills. Would person R have suicidal thoughts? 1. No 2. Slight 3. Moderate 4. Strong	1	1	1	1	2.8
28	Person R cannot find work and the family is destitute. Person R's family is about to be turned out onto the street by debt collectors. Would person R have suicidal thoughts? 1. No 2. Slight 3. Moderate 4. Strong	1	1	1	1	3.78
29	Person S has a 3 year old child with terminal (will be fatal) cancer. Would person S have suicidal thoughts? 1. No 2. Slight 3. Moderate 4. Strong	0.2	0.6	1	0.9	0.48
30	Person U is convicted of rape and murder, and has been sentenced to life in jail without parole. Would person U have suicidal thoughts? 1. No 2. Slight 3. Moderate 4. Strong	0.8	0.9	1	1	3.78
31	Person V is the spouse of Person U (the rapist-murderer in question 33). Would person V have suicidal thoughts? 1. No 2. Slight 3. Moderate 4. Strong	0.6	0.9	0.9	0.9	2.1
32	Person W had always been popular. However, since winning a prize, Person W has been subjected to a sustained, malicious web campaign, including accusations of conceit, sexual deviance and dishonest acts. Would person W have suicidal thoughts? 1. No 2. Slight 3. Moderate 4. Strong	0.6	0.5	0.9	0.8	1.65

### Reliability results

In the reliability study, we examined the two domains of internal consistency and temporal stability. In the internal consistency, 151 respondents completed the questionnaire. Then we calculated Cronbach's alpha and McDonald's Omega coefficients as 0.94 and 0.943, respectively, according to which the questionnaire has acceptable reliability in terms of internal consistency. In the temporal stability, we asked the 30 respondents to fill in the questionnaire 2 weeks later. The correlation was measured using the retest method, using intraclass correlation with a two-way mixed model and consistency type. The ICC was 0.998 (95% CI: 0.997–0.999). Furthermore, we conducted a paired *T*-test in which the test and retest scores means were 55.73 (SD = 21.36) and 56.53 (SD = 20.73), respectively (*p*-value = 0.001). Therefore, it can be concluded that this questionnaire has acceptable reliability in terms of time stability.

### Construct validity results

We performed confirmatory factor analysis using R software version 4 that confirmed that the questionnaire has one dimension. The results of this study can be seen in Table 4. Only the SRMR index, which should be below 0.08, has a slightly higher value, but the other recommended indices are within the significant range. Each question in the fitted model also had significant coefficients.

**Table 4:** Confirmatory factor analysis.

**Measure's name**	**Value**	Cut-off for good fit^*^
χ^2^/df	1.269	< 3
CFI	0.978	CFI ≥ 0.90
TLI	0.986	TLI ≥ 0.95
RMSEA	0.042	RMSEA < 0.08
SRMR	0.092	SRMR < 0.08

^*^Obtained from this article (33).

## Discussion

This study investigated the validity and reliability of the “Social Attitudes to Suicide dealing with Predicaments” questionnaire. We prepared the final Persian version of the questionnaire according to the obtained results, which has acceptable validity and reliability.

The Predicaments questionnaire was designed to assess the community's attitude toward suicide and the impact of specific circumstances on decision-making for suicide. Therefore, it can be used to compare social groups. In this questionnaire, the respondents confront imaginary characters facing a set of challenging situations (28). We asked the respondents to decide whether these characters experience suicidal thoughts and to what extent. Afterwards, we scored the answers, and each person's total score shows the suicidal attitude for each respondent.

Social attitudes link crises and life problems to suicide, which are severe and unfavorable conditions, such as severe mental disorders and terminal diseases. In such conditions, the person considers death the only solution (34). However, these conditions and problems do not necessarily lead the individual to attempt suicide. As stated by the interpersonal-psychological theory of suicidal behavior, suicide will not be desired or attempted unless an individual loses their belonging to close others (thwarted belongingness [TB]) or feels their existence is a burden to others (perceived burdensomeness [PB]). The coexistence of TB and PB is highly associated with suicidal ideation and desire (35). At the same time, the level of adaptability of people in dealing with problems and crises is different. Hence, the response of a person with a low level of adaptability to a simple problem can lead to suicide. To measure individuals' suicide desires, specific instruments have been introduced, among which the Interpersonal Needs Questionnaire (INQ-B) stands out. INQ-B directly measures TB and PB, making it a highly appropriate tool to assess suicidal ideation in individuals, reporting how likely that individual desires to attempt suicide (36). As a different approach, in our study, the Predicaments questionnaire aims to assess the attitudes of the society, rather than the individuals themselves, on whether individuals facing life crises, e.g., loss of belongingness, has the right to desire suicide. Thus, the high scores of the Predicaments questionnaire in a population indicate the extent to which the population will allow a person in crisis to have the right to consider suicide as the solution to that problem.

Moreover, it ultimately predicts a high suicide rate in that group. This questionnaire's scores show a direct relationship between suicide and suicide rate in a group or population. Hence, one may use this tool to prevent suicide worldwide (28).

Most questionnaires measuring attitudes toward suicide are designed for a specific social, age, or occupational group and, in general, for a particular target group. A major problem in measuring attitudes toward suicide is the mental nature of attitudes and their variability over time (26).

The attitude of society plays a vital role in one's beliefs in justifying oneself in ending their life (37). An effective way to prevent suicide is to increase awareness of attitudes and taboos about suicide (38). Various studies have identified deterrents to suicide, including religious belief and anxiety about death (39).

A critical feature of the Predicaments questionnaire that prioritizes it over other similar ones is that it assesses the legitimacy of suicide by using empathy and judgmental emotions; the greater the complexity of the predicaments, the more people's attitudes soften toward suicide, and the more severe the predicament is, the more judgmental emotions are supposed to arise (28).

### Suggestions and limitations

We suggest adding questions specific to the cultural context of Iranian society to this questionnaire in future studies. We recommend measuring individuals' attitudes toward suicide on a large scale in different parts of Iran and comparing the results between diverse populations with different suicide rates. Higher scores of this questionnaire are expected to be recorded in communities with higher suicide rates. Obviously, by conducting large-scale studies at the international level, one might obtain sufficient evidence to determine the underlying suicide risk factors.

One of the limitations of this study is the small sample size. Moreover, this small sample size consists of people from a limited age range. Thus, the results are not generalisable to all people. Another limitation was that it had not measured the relationship between the questionnaire and other questionnaires measuring suicide in order to measure the convergent validity.

## Conclusions

Overall, based on the results of this study, it is confirmed that the Persian version of the Predicaments questionnaire has acceptable content and face validity, temporal stability, and internal consistency. Therefore, this questionnaire can be used in future research on Iranian society.

Suicide is positively influenced by society's social and cultural attitudes that to what extent they assume it as an option in response to the hardships of life. This study proved that this questionnaire is a novel tool capable of measuring people's attitudes toward suicide, hence a proper measure to implement suicide prevention efforts.

## Data availability statement

The raw data supporting the conclusions of this article will be made available by the authors, without undue reservation.

## Ethics statement

The studies involving human participants were reviewed and approved by Ethics Committee of the Iran University of Medical Sciences. The patients/participants provided their written informed consent to participate in this study.

## Author contributions

MS and AR designed the study. AR, AK, and MS made substantial contribution in data gathering. AR, LJ, and YP conducted the analysis. AR drafted the manuscript. SKM, SAM, and AK reviewed the manuscript. AR and MS contributed to the finalising of the manuscript. All authors read and approved the final manuscript.
